# Predation on Rose Galls: Parasitoids and Predators Determine Gall Size through Directional Selection

**DOI:** 10.1371/journal.pone.0099806

**Published:** 2014-06-11

**Authors:** Zoltán László, Katalin Sólyom, Hunor Prázsmári, Zoltán Barta, Béla Tóthmérész

**Affiliations:** 1 Hungarian Department of Biology and Ecology, Babeş-Bolyai University, Cluj-Napoca, Romania; 2 Ecological Department, University of Debrecen, Debrecen, Hungary; 3 MTA-DE “Lendület” Behavioural Ecology Research Group, Department of Evolutionary Zoology, University of Debrecen, Debrecen, Hungary; 4 MTA-DE Biodiversity and Ecosystem Services Research Group, University of Debrecen, Debrecen, Hungary; Dauphin Island Sea Lab, United States of America

## Abstract

Both predators and parasitoids can have significant effects on species’ life history traits, such as longevity or clutch size. In the case of gall inducers, sporadically there is evidence to suggest that both vertebrate predation and insect parasitoid attack may shape the optimal gall size. While the effects of parasitoids have been studied in detail, the influence of vertebrate predation is less well-investigated. To better understand this aspect of gall size evolution, we studied vertebrate predation on galls of *Diplolepis rosae* on rose (*Rosa canina*) shrubs. We measured predation frequency, predation incidence, and predation rate in a large-scale observational field study, as well as an experimental field study. Our combined results suggest that, similarly to parasitoids, vertebrate predation makes a considerable contribution to mortality of gall inducer larvae. On the other hand, its influence on gall size is in direct contrast to the effect of parasitoids, as frequency of vertebrate predation increases with gall size. This suggests that the balance between predation and parasitoid attack shapes the optimal size of *D. rosae* galls.

## Introduction

The dynamics of populations are generally affected by influences from all levels of their food-webs. As a consequence, it can be difficult to understand population processes without taking full account of the interactions at every level. Galls are growths on plants usually caused by invertebrates as insects or mites. Their morphological diversity is mostly explained as an adaptation to lower the impact of natural enemies as insect parasitoids. The effects of insect parasitoids on abundances of plant galls have been studied extensively [1]. The influence of predators, however, has received comparatively little attention. It is thought that invertebrate predators’ impact is diminished because of the galls’ architecture. In galls induced by gall wasps, for instance, the gall chamber’s walls tend to become thick and woody, and hence impenetrable for invertebrates, as they mature. However, the same may not be true for vertebrate predators.

Vertebrate predators of plant galls can be small mammals like squirrels and mice [2], [3] or birds such as woodpeckers and chickadees [4]–[6]. Predation by birds has been investigated in the case of gall inducer species like *Eurosta solidaginis* [6], [7], *Rabdophaga strobiloides* [8], *Giraudiella inclusa* [9], *Asteromya carbonifera* [10]. In a recent study, Schönrogge et al. quantified bird predation on invading insect galls [11]. However, the impact of vertebrate predation on a native gall ecosystem, was until now not published. Here we look at vertebrate predation in an observational large scale and an experimental field study to assess its impact on rose galls.

The size of the gall created by gall inducing insects seems to be an important life history trait. In the case of *D. rosae*, the body size of gall inducer adults is positively correlated with the size of the gall from which they hatched [12]. Thus, hatching from larger galls can increase the fitness of gall inducers, because the number of eggs laid increases with the size of the female [13], [14]. Gall size also influences fitness through larval mortality. With increasing gall size, larval mortality, parasitism rate and abortive hatching decreases while hatching success increases [10], [15]. A possible explanation for this effect is that larvae in smaller galls have to face more frequent parasitoid attacks: they are in easier reach of the parasitoids’ ovipositors as smaller galls have thinner outer walls. These observations suggest that larger galls are more secure and therefore, gall wasps inducing larger galls are more successful, in term of avoiding parasitoids, than those inducing smaller ones [15].

For predators, it can be more advantageous to attack larger galls, because they contain more food and can more easily be discovered [16]–[19]. Consequently, survival of whole galls decreases as their size increases [10], [20]. For instance, in the goldenrod ball gall the rate of bird predation depends on the gall size and the stem height [6]. Furthermore, in large galls, competitive interactions between the developing larvae could also be higher [10], [13]. These counteracting selection pressures induced by parasitoids and predators may stabilize gall size at an intermediate size. This balancing process may also explain why the most frequent gall size in *D. rosae* is smaller than that predicted to overcome insect parasitoid attacks [21].

Despite the fact that close interactions between birds and insects are common, they have been not studied frequently in the arthropod herbivore group [22]. Several studies have investigated the effects and outcomes of bird predation on insects and found that the removal of birds from host plants affected by herbivorous insects increased the levels of herbivory [23]–[25]. Nevertheless, in a study on 78 holometabolous species representing the major insect orders, predators of insect herbivores had a smaller effect on their abundances than parasitoids, but a greater one than pathogens. Several investigations have demonstrated that for gall inducers, including *D. rosae*, parasitoid attacks are the most common mortality factor [20], [26]–[28]. The level of predation on galls of *D. rosae*, however, has not been explicitly reported. The fact that levels of (i) parasitoidism and (ii) predation can easily be quantified by identifying parasitoids emerging from galls and visually inspecting destructive gall openings, respectively, provides a unique opportunity to compare the effect of parasitoidism and predation on the population processes of rose gall inducers.

The gall wasp (*Diplolepis rosae*) is the most abundant galling species on *Rosa* shrubs in Europe. *D. rosae* usually induce galls on *R. canina*, but may also do so on other rose species [13], [29], [30]. Females induce multi-chambered galls. They emerge from galls in early spring and lay their clutches in new rose buds within one or two months. The new gall finishes its development in late summer and pupae overwinter within the gall. Parasitoid pressure on *D. rosae* galls is high, as is the frequency of the inquiline species *Periclistus brandtii* [31].

The most abundant parasitoid species on *D. rosae* galls are *Orthopelma mediator*, *Torymus bedeguaris*, *Glyphomerus stigma*, *Pteromalus bedeguaris*. On the other hand, *Caenacis inflexa* is exclusively a parasitoid of the inquiline *P. brandtii*. Other species, such as *Torymus rubi, Eupelmus urozonus, Eupelmus vesicularis, Eurytoma rosae*, can be parasitoids of both the gall inducer and inquiline [32], [33].

Here we investigate the following questions: (i) Is there any preference by vertebrate predators towards larger *D. rosae* galls? (ii) How does gall inducer mortality caused by vertebrate predation compare to that caused by insect parasitoids? If the predation on galls by vertebrates has a significant effect on the success of gall inducers then this may explain why galls of small sizes exist, even in the face of the high parasitoid pressure that has been shown previously for our study populations [21]. We investigated these questions with both a large-scale observational field study, as well as an experimental field study.

## Materials and Methods

### Ethics Statements

All study sites were located in public areas and no specific permission was required for these sites and experiments. Field studies did not involve endangered or protected species. During the study no invasive collecting and sampling methods were used. We evaluated the effect of vertebrate predators based on their feeding signs.

### Study Sites

Data were collected in Hungary and Romania between 2008 and 2012 from semidry and dry pastures with high rose shrub abundances. Predation of rose galls had also been observed in previous years at all collecting sites.

For the observational study, two distant sites were chosen, one near Cluj-Napoca, Romania (*site 1a*, N 46.770208, E 23.493241), and the other near Derecske, Hungary (*site 2*, N 47.332944, E 21.561032) to obtain coverage on a large spatial scale. Both sites consisted of semidry and dry pastures close (cca. 100–300 meters) to oak and hornbeam-oak forests, with shrub species *Prunus spinosa* and *Crataegus* spp. in addition to the dominant *Rosa canina*.

**Figure 1:**
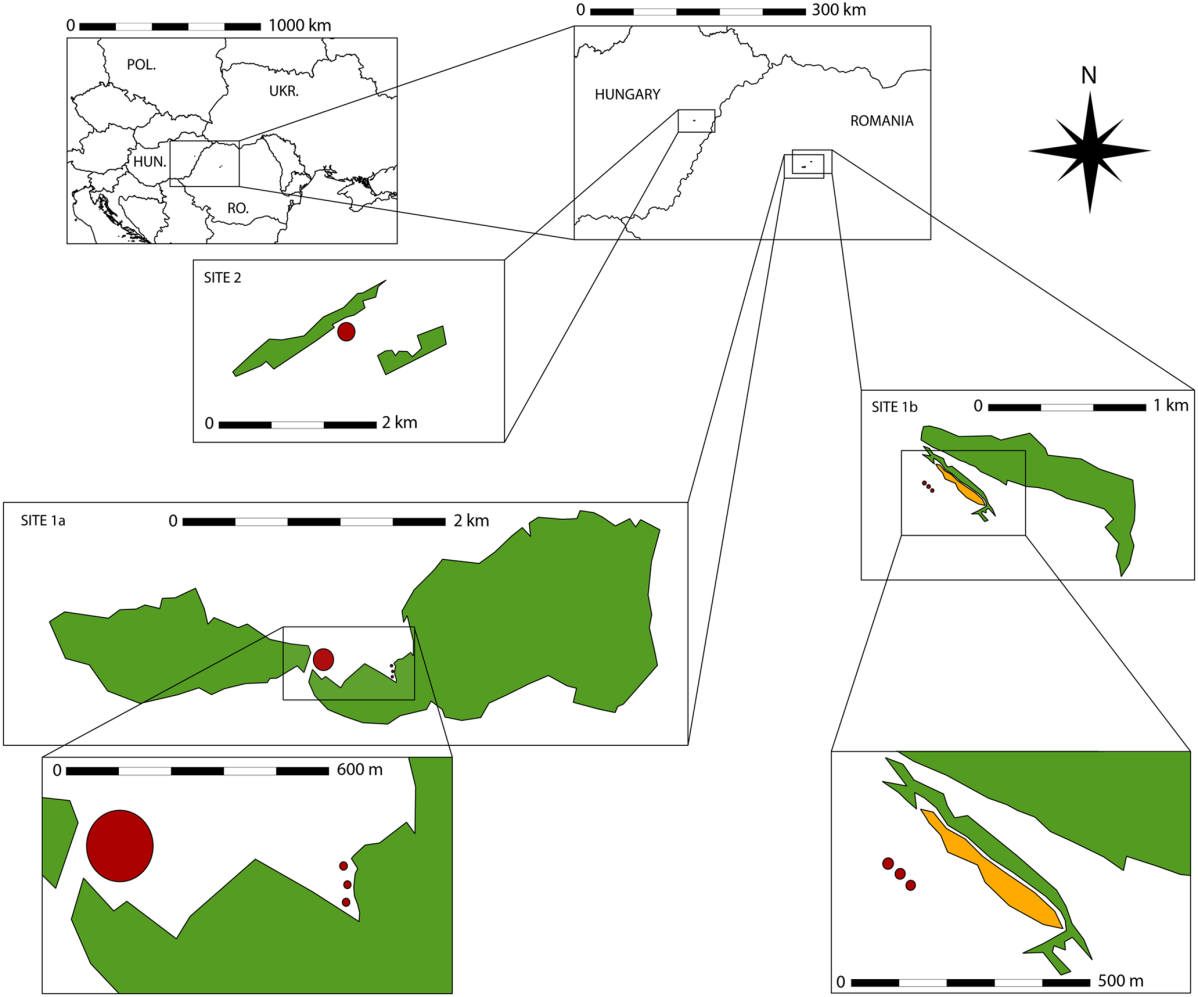
Map of the study sites. Large red circles show the locations of observational study sites. Small red circles show the locations of the experimental study. Green polygons are forest patches around study locations. Orange polygon is a larger hillock between the study site and woody vegetation.

For the gall predation experiment, two sites were chosen in the vicinities of Cluj-Napoca, Romania (Figure 1). One was one of the observational study sites (*site 1a*), the other (*site 1b*, N 46.836305, E 23.623328) was 13 kilometres from *site 1a*. *Site 1b* was far from any secondary hornbeam-oak forests, and the closest deciduous-only habitat was an old orchard 1.5 kilometers away; rather, in the close vicinity (cca. 100–300 meters) there were plantations of *Pinus nigra* mixed with *Elaeagnus angustifolia* and *Pyrus pyraster*.

### Gall Predation Experimental Setup

At both *site 1a* an*d si*t*e 1b* we chose three patches of shrubs, each consisting of three shrubs of similar size. At *site 1a* the shrubs were 10–20 meters from the edge of the hornbeam-oak forest, while at *site 1b* the closest trees were at a distance of 100–120 meters from the chosen shrubs. At both sites, the following treatments were randomly allocated among the shrubs within each patch. We placed ten large (diameter: mean = 3.08 cm, SD = 0.52) galls on one shrub, and ten small (diameter: mean = 1.66 cm, SD = 0.43) ones on another, while the third shrub received five small and five large galls (these galls were collected from shrubs outside the experiment patches, and the shrubs in the experiment had no galls beforehand). Hence, 30 galls were placed in each shrub patch, giving 90 galls at each site. Galls were bound with thin wires of the same color as the shrub shoots at approximately at the same height, between 1.5–2 meters high. Galls were placed on shrubs in December, and collected in late March to early April. All experimental galls yielded living hymenopteran adults belonging to the community of the gall inducer, allowing us to assume that they were viable. The experiment was run at both sites in 2009, 2010, and 2011, and only at *site 1a* in 2012. Emerging specimens were only collected in 2011; in other years, we assumed the galls’ viability based on freshness of gall samples. Placed experimental galls were numbered according to a combination of site name, shrub patch number, shrub type (with small, large and combined gall sizes), and gall number.

The galls placed on the experimental shrubs in 2011 were removed in spring 2012 and stored individually in plastic cups, with cellophane covers allowing air inside, and kept under standard laboratory conditions [33]. Emerged specimens were separated, then stored in 70% ethanol until identification.

Insect parasitism was assessed through identification of emerged parasitoids, while vertebrate predation was assessed through visual identification of gall openings that appeared during the winter. Insect predation on gall chambers of *D. rosae* is not possible after the matured, both because of the hardness of the gall walls and because of the timing of maturation, which occurs at the end of the vegetation period. In addition, the openings observed on the exposed galls were the result of hard punctures with presumably conical or tapered beaks, and did not resemble the sawing pattern produced by the incisor teeth of rodents.

### Statistical Analyses

For each gall the level of predation was characterized by the following dependent variables: (i) presence of predation signs on galls (hereafter, gall predation incidence) (ii) the number of opened gall chambers per gall (hereafter, gall predation), and (iii) the proportion of opened chambers to the estimated number of the gall chambers (hereafter, gall predation rate). Gall parasitism rate, i.e. the ratio of the number of total emerged parasitoids and the estimated number of chambers for each gall was also included in the analyses as a dependent variable. The estimation of the number of gall chambers was based on a power law relationship between gall diameter and gall chamber number established in a previous study [33]; the estimated chamber number was rounded to the nearest integer.

For the observational field study, we entered years, sites, and ID of shrubs in the generalized linear mixed effect models (GLMM) as random factors. Gall diameter was used as a fixed factor. Gall diameter was calculated as the average of the largest three orthogonal diameters of each gall [33].

For the experimental study, years, sites, and ID of shrubs were used in the GLMM as random factors. As fixed factors we entered the gall diameter, shrub treatment type (with large, mixed and small galls), and the site (*site 1a* and *site 1b*). To compare the effects of gall predation and parasitism we entered mortality rate as a dependent variable and the cause of mortality (i.e. predation or parasitism) as a fixed factor, and we used gall diameter as a covariate. This last analysis was performed only on the experimental data from 2011–2012.

The analyses were performed in the statistical programming language *R* [34]. For the observational and experimental studies we used GLMMs with different error distributions. For the gall predation data we used a zero inflated negative binomial GLMM. For the gall predation incidence and gall predation rates we used a binomial GLMM. In the case of gall predation rates we set up a pair of “success” and “failure” variables [35]: successes were the gall predation data; failures were the number of chambers minus gall predation. For the comparison of gall predation and parasitism rates we used a GLM with quasibinomial error distribution and a logit link function. The quasibinomial error distribution was due to the overdispersion of the outcome variable. The used R packages were “glmmADMB” [36], “lme4” [37], “MASS” [38] for analyses and “ggplot2” [39] for graphics. For presentation of parasitoid attack and vertebrate predation rates as the function of gall diameters we used non-parametric locally weighted polynomial regression [36].

## Results

### Gall Size

**Figure 2:**
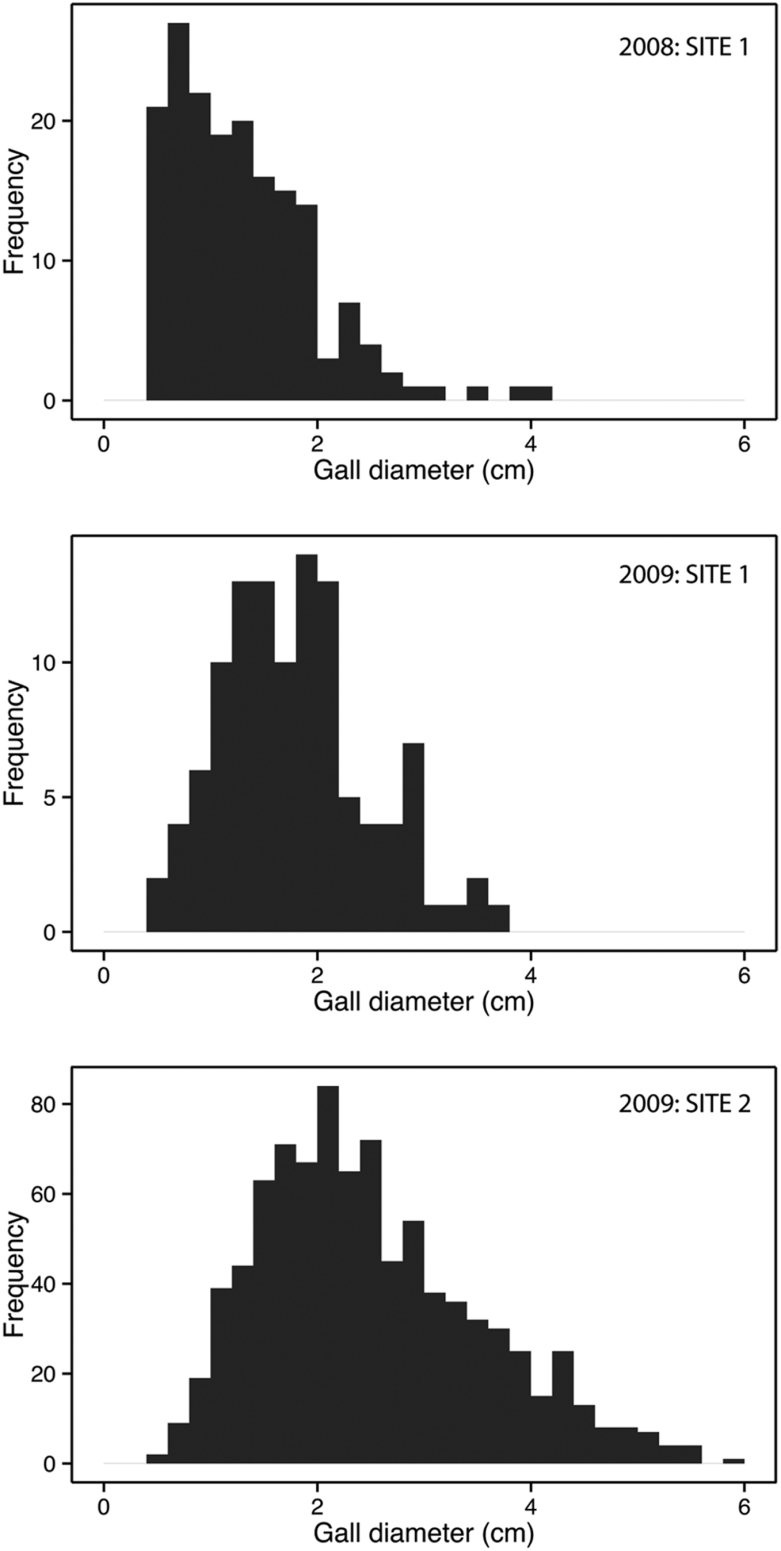
Frequency distribution of gall sizes for theobservational study.

The sizes of galls collected in the observational field study had right-tailed frequency distributions (Figure 2). At *site 1a* in 2008 the median gall size was 1.17 cm (IQR = 0.89, min = 0.47, max = 4.06, N = 175), and in 2009 it was 1.75 cm (IQR = 0.83, min = 0.57, max = 3.73, N = 880). At *site 2* in 2009 the median gall size was 2.30 cm (IQR = 1.43, min = 0.50, max = 5.87, N = 110). For the experimental study the statistics of the gall sizes are given in Table 1.

**Table 1:** *Diplolepis rosae* gall size (cm) descriptive statistics for the experimental study.

year	site	shrub type	median	IQR	min.	max.
2009	*site 1a*	large	2.71	0.43	1.59	3.78
		mixed	2.09	1.46	0.67	3.50
		small	1.82	0.64	0.83	2.45
	*site 1b*	large	2.97	0.89	1.76	3.81
		mixed	2.43	1.94	1.18	5.45
		small	2.06	0.61	1.50	3.07
2010	*site 1a*	large	3.72	0.63	2.55	4.78
		mixed	2.52	1.39	1.54	4.70
		small	2.20	0.40	1.50	2.87
	*site 1b*	large	2.65	0.83	1.29	4.41
		mixed	2.60	1.34	0.88	4.26
		small	1.51	0.74	0.55	2.78
2011	*site 1a*	large	3.17	0.59	1.59	4.27
		mixed	2.35	1.48	0.66	3.81
		small	1.59	0.45	1.05	2.14
	*site 1b*	large	2.65	0.47	1.53	3.93
		mixed	2.15	1.33	0.82	4.87
		small	1.62	0.64	0.90	2.28
2012	*site 1a*	large	3.26	0.41	2.49	4.78
		mixed	2.07	1.60	1.00	4.25
		small	1.55	0.58	0.99	2.32

In the experiment we used in total N = 630 galls, N = 90 on each site in each year. Shrub treatment types: large had only large galls, mixed had half large and half small, and small had only small galls.

### Observational Study: Predation

At *site 1a* and *site 2*, out of a total number of N = 1165 galls N = 245 (21%) had been opened by predators. The opened galls were observed on N = 52 out of N = 166 (31%) rose shrubs (Table 2). At *site 1a*, the median number of opened gall chambers of predated galls was 6 (IQR = 8, min = 1, max = 50), while at *site 2* it was 8 (IQR = 11, min = 1, max = 60). The incidence of predation was 21% when both years and sites were combined; at *site 1a* it was 23% and at *site 2* it was 20%. After controlling for year, site and shrub ID, all dependent variables characterizing predation increased with gall size (gall predation: negative binomial GLMM, estimate = 0.95, SE = 0.11, z = 8.55, p<0.001; incidence of gall predation: binomial GLMM, estimate = 1.29, SE = 0.25, z = 5.11, p<0.001; and predation rate: quasibinomial GLMM, estimate = 0.17, SE = 0.05, df = 1050, t = 2.9, p = 0.002).

**Table 2:** Vertebrate predation frequencies for *Diplolepis rosae* galls in the observational study.

year	site	shrubs		galls	
		predated	not predated	predated	not predated
2008	*site 1a*	13	1	37	138
2009	*site 2*	30	82	179	701
2009	*site 1a*	9	31	29	81
sum		52	114	245	920
percent		31.33	68.67	21.03	78.97
total		166		1165	

### Experimental Study: Predation

**Figure 3:**
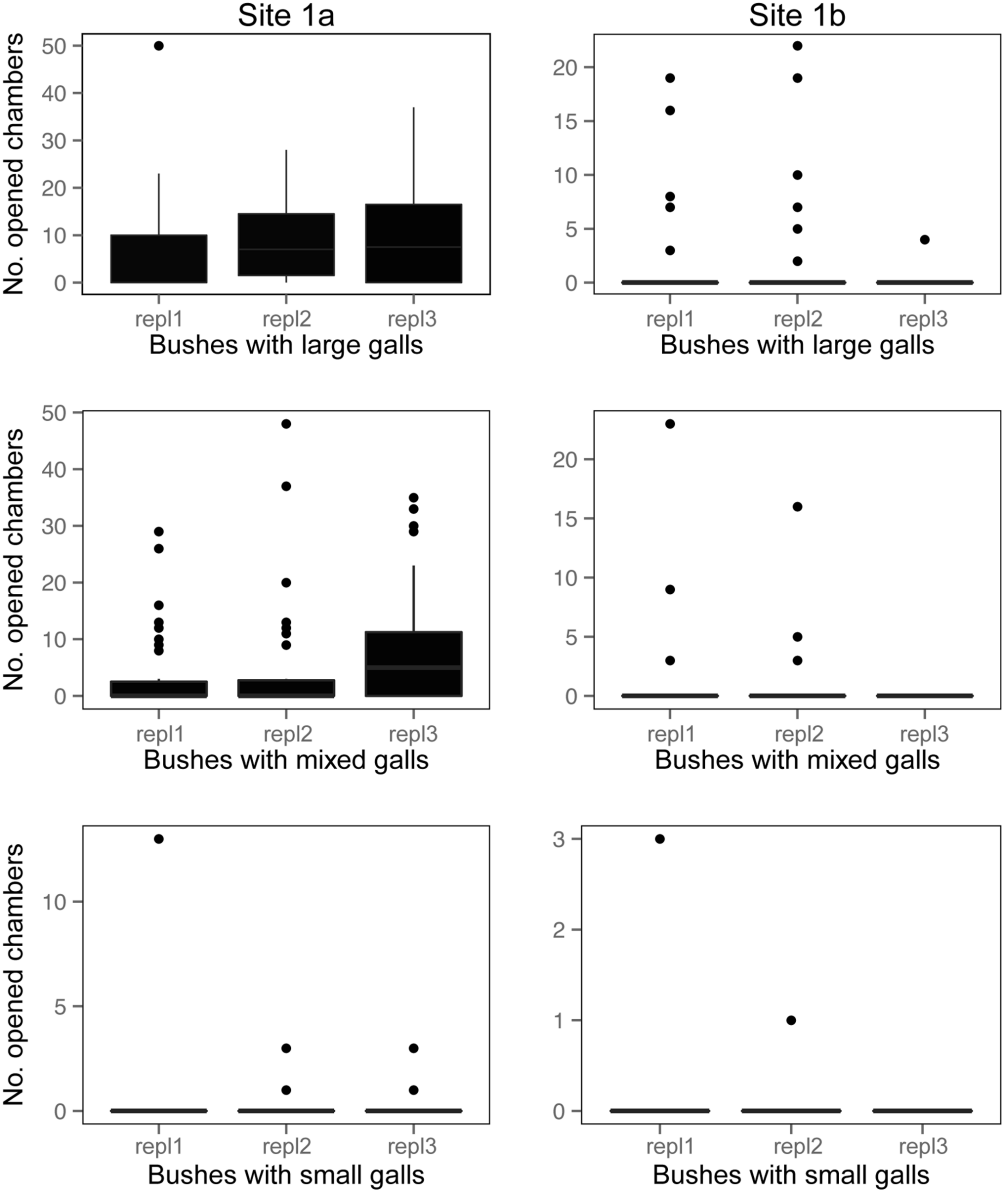
Frequencies of vertebrate predation (opened gall chambers) in the experimental study. Pooled yearly replicates are shown separately for the two experimental sites. Boxes show medians, 1^st^ and 3^rd^ quartiles, error bars represent minimum and maximum values. Dots represent outliers.

**Figure 4:**
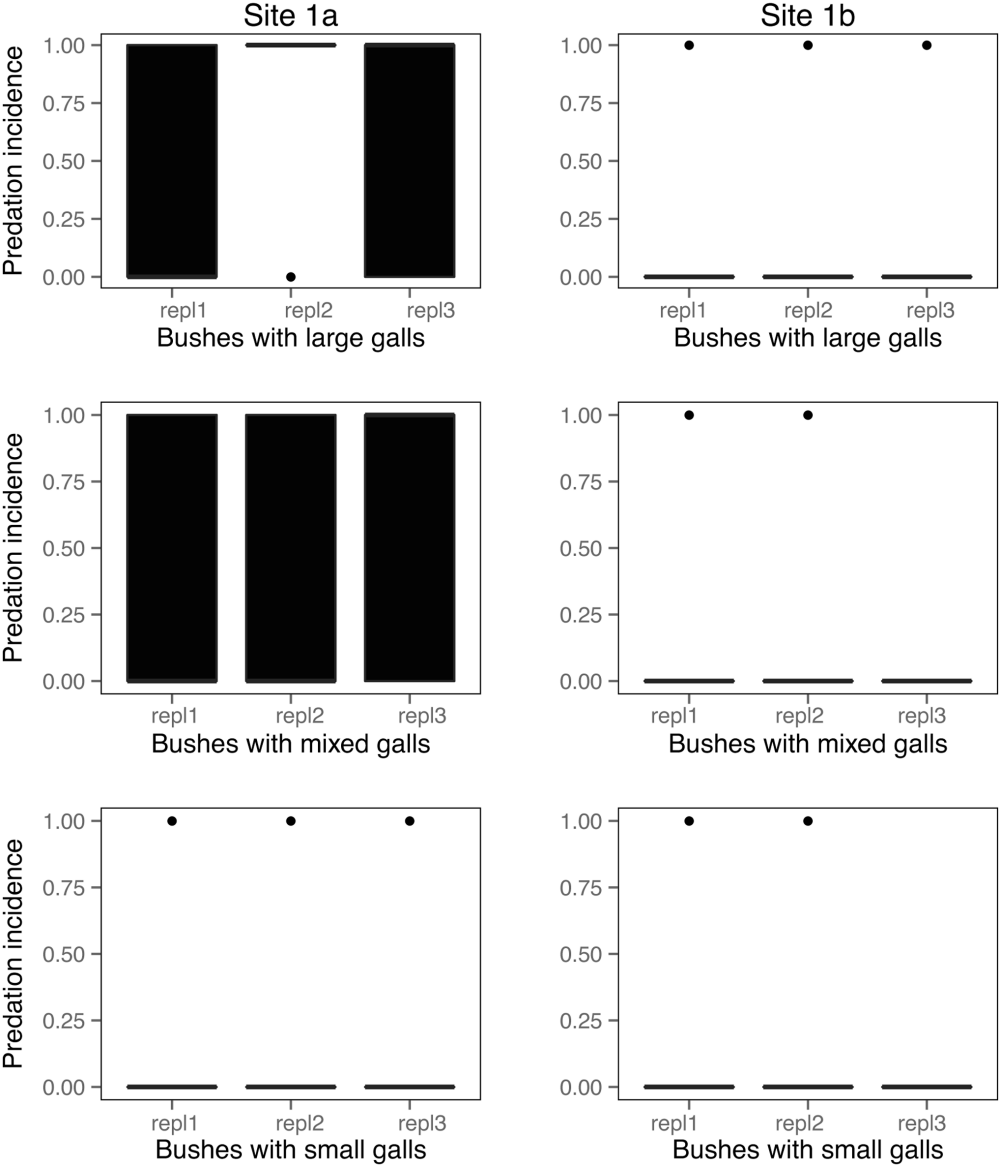
Incidences of vertebrate predation (galls opened or not) in the experimental study. Pooled yearly replicates are shown separately for the two experimental sites. Boxes show medians, 1^st^ and 3^rd^ quartiles, error bars represent minimum and maximum values. Dots represent outliers.

**Figure 5:**
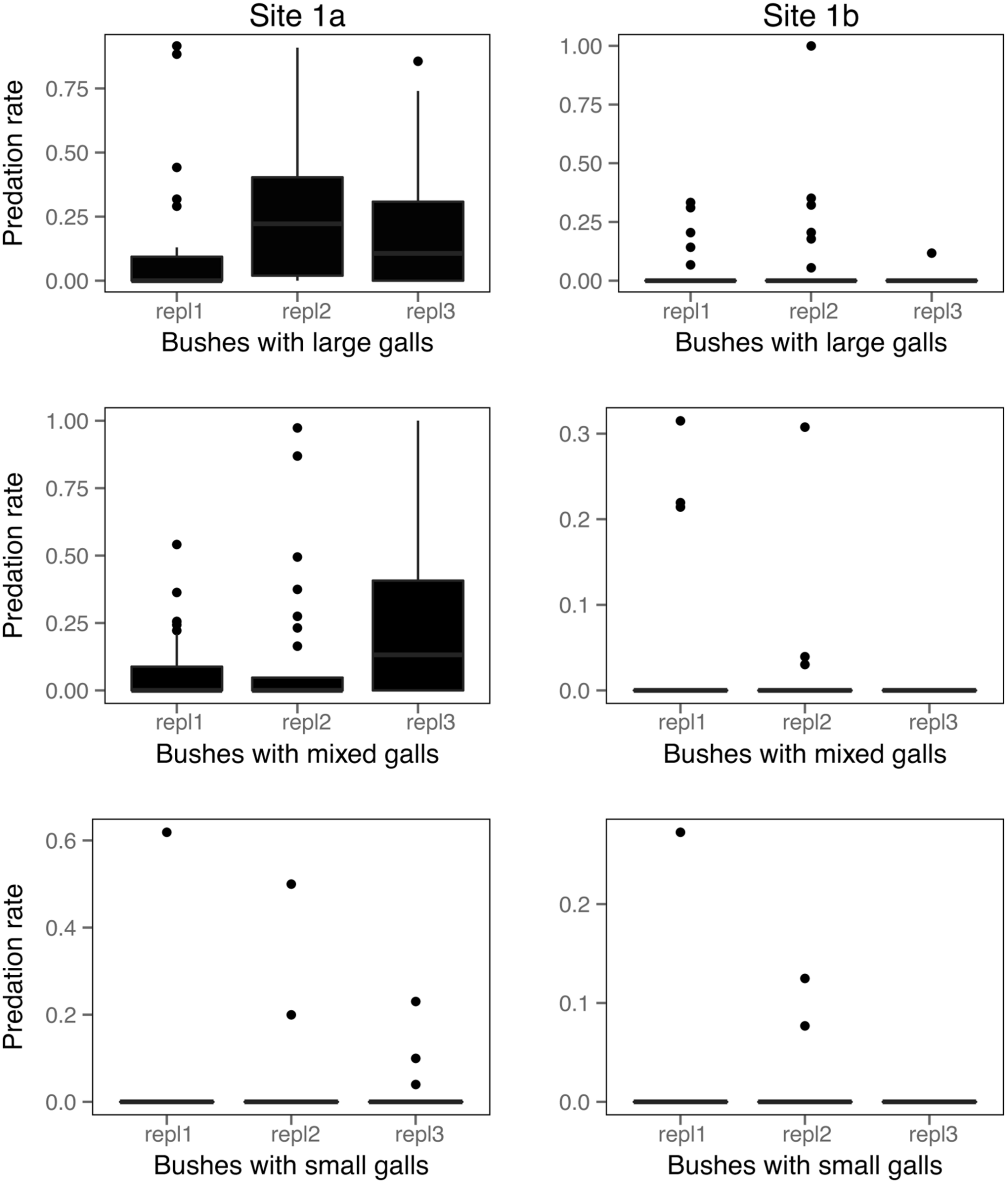
Rates of vertebrate predation in the experimental study. Pooled yearly replicates are shown separately for the two experimental sites. Boxes show medians, 1^st^ and 3^rd^ quartiles, error bars represent minimum and maximum values. Dots represent outliers.

At the two experimental study sites (*site 1a* and *site 1b*), out of a total number of N = 630 galls N = 159 (25.23%) had been opened by predators. The opened galls were observed on N = 14 rose shrubs out of N = 18 (77.77%) (Table 3). The largest number of opened gall chambers was at *site 1a* on shrubs with large galls, and the smallest at *site 1b* on shrubs with small galls (Figure 3). The pattern was similar in the case of the incidence of predation (Figure 4) and predation rates (Figure 5).

**Table 3:** Vertebrate predation frequencies for *Diplolepis rosae* galls in the experimental study.

			No. opened galls				
year	site	shrub type	1^st^ Q	median	3^st^ Q.	sum	total
2009	*site 1a*	large	2	7	8	18	
		mixed	3	4	5.5	14	
		small	0	0	0.5	1	33
	*site 1b*	large	0	0	0.5	1	
		mixed	0	0	0	0	
		small	0	0	0.5	1	2
2010	*site 1a*	large	5	10	10	25	
		mixed	4	5	7.5	19	
		small	0	1	1	2	46
	*site 1b*	large	0	0	0	0	
		mixed	0	0	0	0	
		small	0	0	0	0	0
2011	*site 1a*	large	6	9	9.6	22	
		mixed	3	6	6.5	13	
		small	0	0	0.5	1	36
	*site 1b*	large	2	5	5.5	13	
		mixed	1	2	3.5	8	
		small	0	0	1	2	23
2012	*site 1a*	large	1	1	1.5	4	
		mixed	0	5	5	10	
		small	0	2	2.5	5	19

In the experiment we used in total N = 630 galls, N = 90 on each site in each year. Shrub types: large had only large galls, mixed had half large and half small, and small had only small galls.

Similarly to the observational study, after controlling for year, site and shrub ID, all dependent variables characterizing predation increased with increasing gall size (gall predation: negative binomial GLMM, estimate = 0.85, SE = 0.17, z = 4.78, p<0.001; the incidence of gall predation: binomial GLMM, estimate = 1.09, SE = 0.18, z = 5.89, p<0.001; predation rate: quasibinomial GLMM, estimate = 0.36, SE = 0.11, df = 521, t = 3.21, p = 0.001).

Furthermore, the difference in predation level between treatment types was only significant between shrubs containing large and small galls (gall predation: negative binomial GLMM, estimate = −2.10, SE = 0.51, z = −4.12, p<0.001; incidence of gall predation: binomial GLMM, estimate = −1.56, SE = 0.50, z = −3.12, p<0.001; predation rate: quasibinomial GLMM, estimate = −1.88, SE = 0.62, df = 521, z = −3.00, p = 0.002). Shrubs with mixed gall treatments showed no difference compared to those with large galls.

Finally, there was a highly significant difference in predation between the two sites (*site 1a* and *site 1b*) (gall predation: negative binomial GLMM, estimate = −2.05, SE = 0.44, z = −4.64, p<0.001; the incidence of gall predation: binomial GLMM, estimate = −2.25, SE = 0.31, z = −7.26, p<0.001; predation rate: quasibinomial GLMM, estimate = −2.13, SE = 0.26, df = 521, t = −8.30, p<0.001).

### Extent of Parasitism and Predation Rates

From the galls placed on the experimental shrubs in winter 2011 and re-collected in spring 2012, 649 hymenopteran specimens emerged in the case of *site 1a* and 419 specimens in the case of *site 1b* (Table 4). From *site 1a* 28.22% of the gall chambers yielded parasitoids and inquilines, while from *site 1b* it was 51.77%. Gall predation was 57.80% at *site 1a*, and 43.07% at *site 1b*. Consequently, the reproductive success of the gall inducer *D. rosae* was 13.97% at *site 1a* and 5.16% at *site 1b*.

**Table 4:** Numbers of gall chambers predated by vertebrates, as well as numbers of emerged gall inducers, inquilines and parasitoid specimens in the experimental study.

	*site 1a*	*site 1b*
No. opened chambers by predators	889	317
*Diplolepis rosae*	215	38
No total parasitoids and inquilines	434	381
*Periclistus brandtii*	21	3
*Orthopelma mediator*	266	208
*Torymus bedeguaris*	10	29
*Glyphomerus stigma*	86	56
*Pteromalus bedeguaris*	45	37
*Caenacis inflexa*	1	24
*Eurytoma rosae*	3	21
*Eupelmus urozonus*	2	3

**Figure 6:**
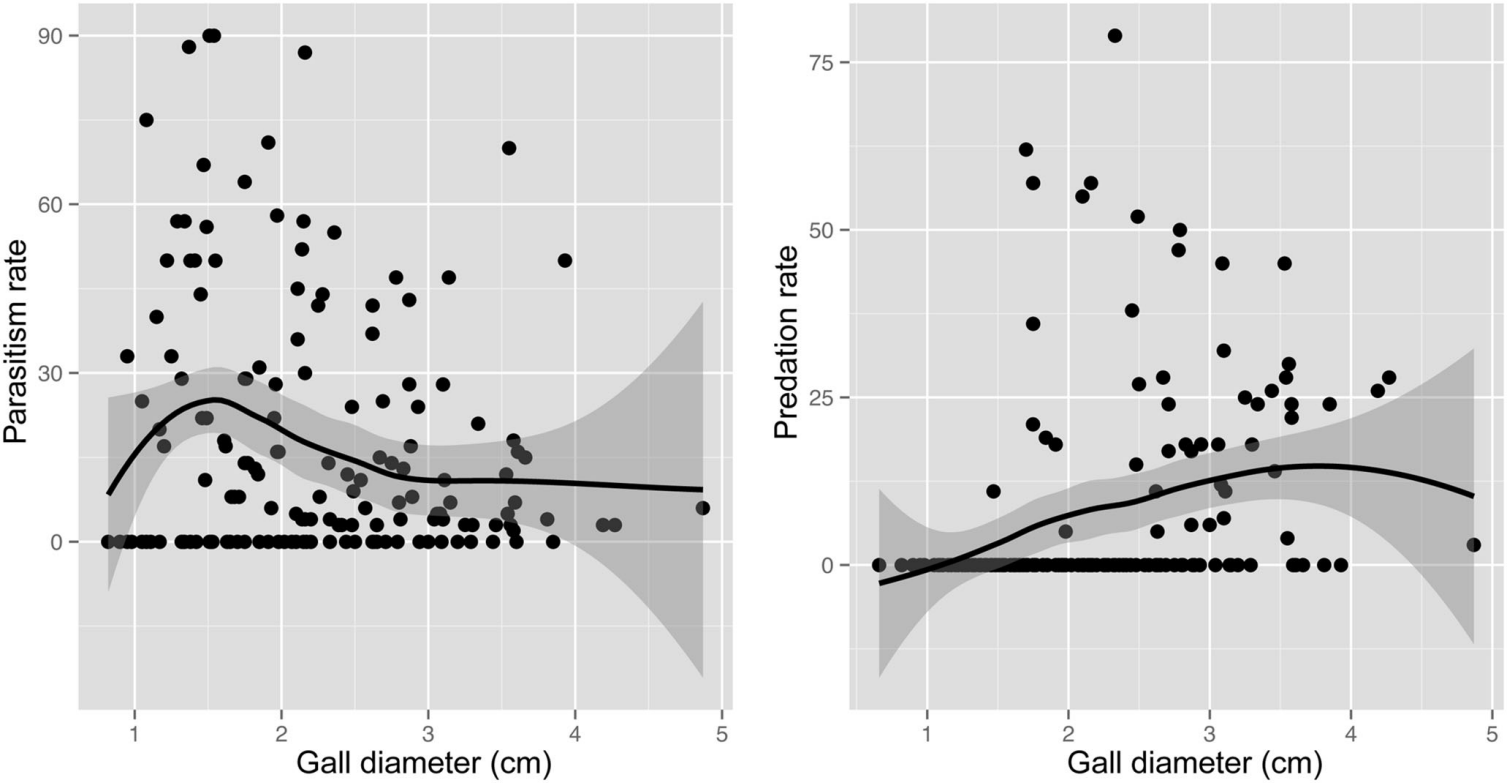
Parasitoid attack and vertebrate predation rates as a function of gall diameters. The fitted curves represent non-parametric locally-weighted polynomial regression curve (loess), the shaded region represents the 95% confidence interval.

The effect of gall size on mortality rate varied according to the causes of mortality (Figure 6; gall size × cause of mortality interaction: quasibinomial ANCOVA, estimate = 0.97, SE = 0.26, z = 4.79, p<0.001). There was a significant negative correlation between parasitism rate and gall size (quasibinomial ANCOVA: estimate = −0.37, SE = 0.15, z = −2.53, p<0.001); in contrast, predation rate and gall size showed a significant positive correlation (quasibinomial ANCOVA: estimate = 0.60, SE = 0.14, z = 4.29, p<0.001).

## Discussion

We observed a remarkable level of vertebrate predation on the galls of *Diplolepis rosae*, for all sites and years. We found that predation is a major contributor to the mortality of the gall inducer. Moreover, the vertebrate predation rate increased with increasing gall size, while insect parasitism decreased. These interactions suggest that the optimal gall size may be the result of a trade-off between avoiding both insect parasitism and vertebrate predation.

Gall size distribution was asymmetric, with predominantly smaller galls in our samples. This was previously documented for *D. rosae* galls collected in the vicinities of our study sites [21]. Previous studies on other species have found similarly shaped distributions of gall size: for instance, large galls are less common than small ones in the cases of *Dryocosmus kuriphilus* [22], *Asteromyia carbonifera* [10] and *Urophora cardui* [37].

In the observational study we found that galls with larger diameters were more frequently attacked by vertebrate predators. Furthermore, our experimental result confirms the preference of vertebrate predators for larger galls, and it also corroborates the findings of studies on other galling systems, which show that bird predation of larger galls is more intensive [6], [18].

Both parasitoids and predators may be responsible for shaping gall size distribution [38]. The preference of predators for large galls is advantageous for small galls; at the same time, parasitoids are more successful at attacking small galls, thus driving galls to become larger [17].

Large galls’ preferential attack by vertebrate predators could occur because vertebrate predators are more likely to notice larger galls thus also predating them more frequently. Evidently, larger galls also provide more food. However, in contrast to our results, another study found that birds preferred small willow pinecone gall midge galls (*Rhabdophaga strobiloides*) [8]. This preference may be explained by considering the amount of energy/time invested in handling food: opening small galls is easier, and birds gain food in a shorter time than if they had chosen large galls instead [19]. In other instances, the pattern can be explained by the larger amount of larvae yielded by larger galls.

The difference in predation levels between shrubs containing large and small galls remained significant, even after controlling for gall size. This implies that large galls were predated disproportionately more and more often than predicted based on their size difference. This result implies an accentuated preference for patches of large galls by the predators of *D. rosae*. The reason for this could be the aggregated concentration of large galls: predators search an area more thoroughly if they find profitable prey, i.e.in large galls [33]. A higher concentration of large galls can also increase the likelihood of them being detected by predators. A positive density dependence of bird attacks also was observed for cynipid galls on oak [16]. Our results also suggest that vertebrate predators are able to discriminate between food rewards from galls of different sizes. In contrast, vertebrate predators of goldenrod ball galls did not seem to discriminate between differently sized galls [2].

**Figure 7:**
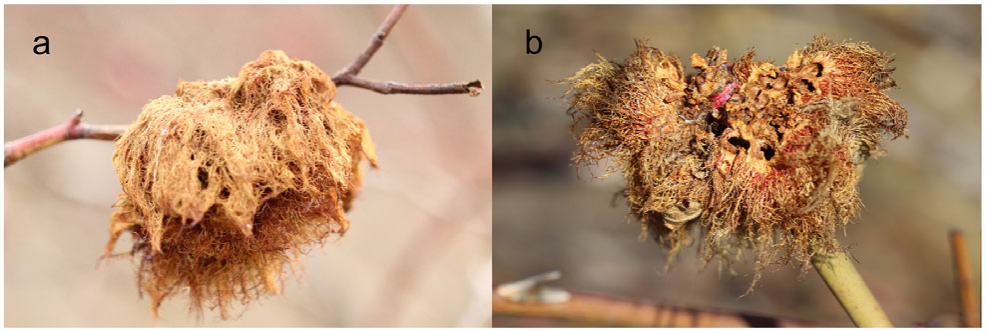
Non-predated and predated rose galls. a) Intact gall, with no signs of predation. b) Heavily damaged gall with chambers opened. Photo credits: László Zoltán.

**Figure 8:**
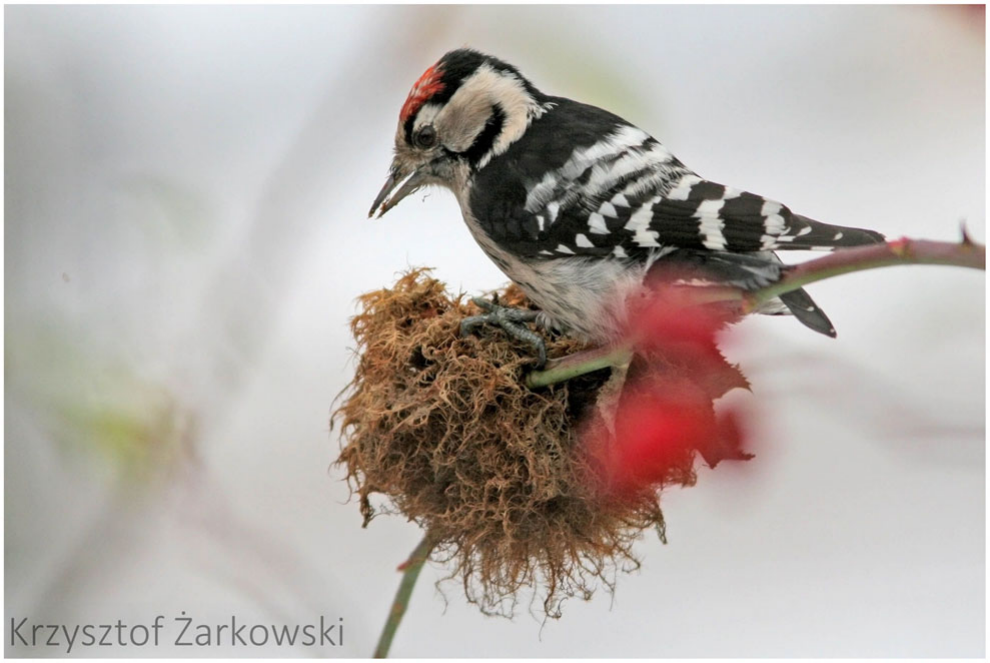
Lesser spotted woodpecker (*Picoides minor*) opening *D. rosae* gall. Photo credits: Krzyszt of Żarkowski.

Larvae of *D. rosae* developing in galls may serve as food for both small mammals and birds. The predation pattern we observed in individual galls, however, indicates that the predators opening rose gall chambers may be birds rather than mammals. The opened galls look like they were punctured using powerful strikes (as from a bird’s beak), resulting a conical pattern with the tip closer to the gall center (Figure 7b), rather than sawed finely from the outside towards the center (as from a mammal’s teeth). We also observed predated *Diplolepis mayri* galls that were close to the ground with their surfaces sawed finely from the outside towards the center. This latter feeding pattern suggests predation by mice rather than birds. Figure 8 shows a lesser spotted woodpecker (*Dendrocopos minor*) opening and feeding with a *D. rosae* gall.

Predator-avoidance behaviour is strongly affected by predator-prey interactions, i.e. if predators are absent preys will lose their aversion towards them, but not rapidly, only in an evolutionary time scale [39]. When predation pressure declines, antipredatory behaviours may decrease in frequency or disappear entirely. This loss or inhibition of ability to act against predators may occur either on evolutionary or ecological timescales [39]. We found a much higher frequency of predation at *site 1a* than site1b. The fact that this site was much closer to the forest edge might indicate that gall predation is somehow more frequent near forests. As the forest cover in the Carpathian Basin has rapidly decreased over the last 500–1000 years [40], one might hypothesise that populations further away from forests may have larger gall size than those closer to forest edges, if predation pressure is lower. Our results only show a correlation, and only at one site, thus they do not constitute solid evidence for this hypothesis. However, this could be a fruitful topic for future research.

To summarize, we found that the galls induced by *Diplolepis rosae* are under rather high pressure from both vertebrate predators and insect parasitoids. The directions of predation and parasitism are opposite with respect to gall size. We suggest that optimal gall size is based on a trade-off between reducing both predation and parasitism rate.
